# Case of Acute Onset Postpartum Paraplegia: Spontaneous Spinal Epidural Hematoma–A Rare Entity

**DOI:** 10.1055/s-0039-1688506

**Published:** 2019-05-22

**Authors:** Praveen Kumar Pandey, Inder Pawar, Harsimar Singh

**Affiliations:** 1 Department of Orthopaedics, ESI-PGIMSR Model Hospital, Basaidarapur, New Delhi, Delhi, India

**Keywords:** SSEH, postpartum, paraplegia, MRI, decompression

## Abstract

**Study Design** Present study is a case report.

**Objective** This study was to present a rare case of acute postpartum paraplegia due to spontaneous spinal epidural hematoma (SSEH).

**Background** SSEH with incidence rate of 0.1 per 100,000 per year is an extremely rare cause of sudden onset neurological deficit in postpartum patients with no predisposing factors or intrapartum factor causing SSEH resulting in paraplegia.

**Material and Methods** We hereby present our case of acute onset postpartum paraplegia which on magnetic resonance imaging (MRI) shows epidural hematoma around thoracic 12 to lumbar 2 (T12–L2) vertebral regions with adjacent cord changes. MRI is the investigation of choice in such cases which helps in proper timely management of patient. We planned the patient for surgical decompression of hematoma.

**Results** Patient showed rapid reversal of neurological symptoms in postoperative period.

**Conclusions** Acute onset postpartum paraplegia in a healthy female with no significant past history, predisposing factors or intrapartum factors may be caused by SSEH and it should be managed on emergency basis as early and proper treatment has an excellent prognosis as seen in our case.

## Introduction

Spontaneous spinal epidural hematoma (SSEH) is an extremely rare entity with incidence of 0.1 per 100,000 every year. 1 This is a rare cause of rapid onset neurological deficit due to which it should be diagnosed early and managed adequately to prevent permanent neurological deficit. Common cause of SSEH includes minor trauma, coagulation abnormalities, vascular malformation, transient vascular hypertension, disc herniation, and idiopathic causes. 2 In review of literature, we didn't find any case of SSEH in postpartum patient leading to acute postpartum paraplegia. We present a case of acute onset postpartum paraplegia with no predisposing factor due to SSEH involving thoracic 12 to lumbar 2 vertebral levels (T12–L2).

## Case Report

**Fig. 1:**
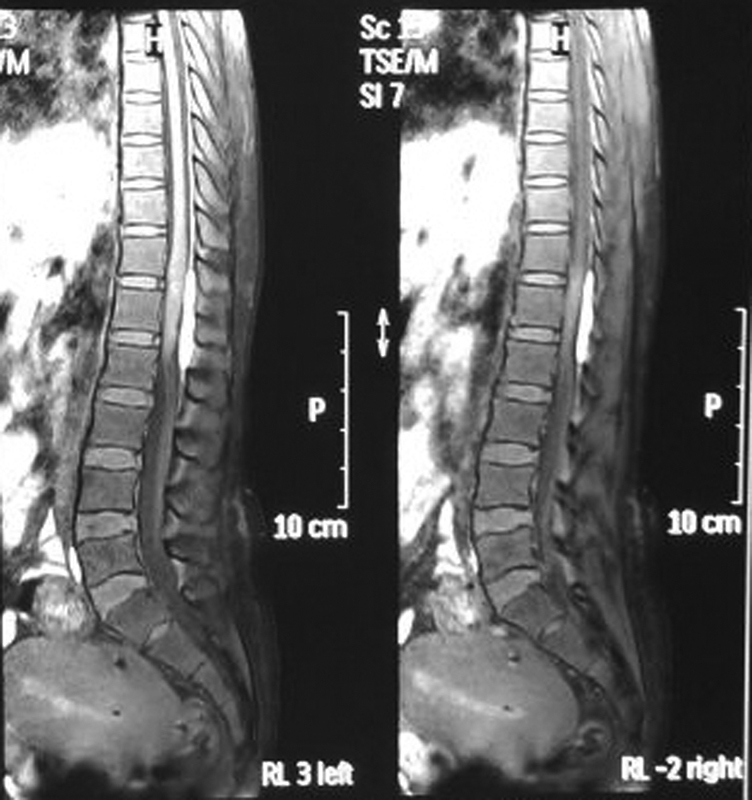
MRI spine sagittal cuts showing extradural broad based ellipsoid lesion measuring 50 × 9 mm along T12–L2 vertebra displacing the dura anteriorly and T1 hyperintensity. L, lumbar; MRI, magnetic resonance imaging; RL, right to left; sc, section; sl, sliceo; T, thoracic; tse, turbo spin echo.

A 29-year-old female patient was referred to our department with acute onset postpartum paraplegia with bowel bladder involvement after delivery of her third child by normal vaginal delivery. Her antenatal course had no significant history with any history of antepartum low-back pain, trauma, drug use, or any physical exertion. On examination, she was conscious, well oriented, and afebrile. Neurological examination showed complete paraplegia (power 0/5 with decreased tone), associated bowel bladder involvement, and absent deep tendon reflexes at knee and ankle joint with complete sensory deficit below the level of L1. All necessary blood investigations including coagulation profile came out to be normal. Patient was planned for magnetic resonance imaging (MRI) of dorsolumbar spine with screening whole spine, which revealed extradural broad based ellipsoid lesion, measuring 50 × 9 mm along T12 to L2 vertebra displacing the dura anteriorly and T1 hyperintensity ( Fig. 1 ), T2 hypointensity with minimal postcontrast enhancement suggestive of early subacute bleed causing mass effect on adjacent cord with areas of altered signal intensity at the level of lesion.

**Fig. 2:**
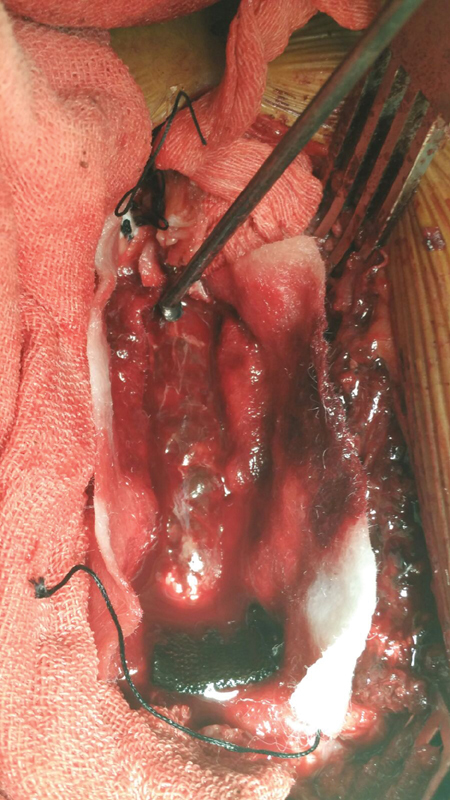
Intraopertive picture after L1 laminectomy showing well-defined firm organized hematoma over the dura which was removed. L, lumbar.

On the basis of clinical evaluation and MRI report, the patient was planned for urgent decompression in emergency operation theater. After preoperative marking of D12–L2 vertebral levels under fluoroscopic guidance, midline exposure done and paraspinal muscles elevated upto medial edge of transverse processes on both sides with the help of cobb's and broad osteotome. Intraoperatively, bilateral complete laminectomy of L1 vertebrae was done with removal of ligamentum flavum, a well-defined firm organized hematoma dark reddish black in color (5 × 1 cm) was seen over the dura which was removed ( Fig. 2 ) and checked for adequate decompression utilizing infant feeding tube. Sample removed was sent for histopathological examination reported as organized hematoma. Postoperatively, patient recovered rapidly in terms of neurological deficit and was discharged on 10th postoperative day with almost complete neurological recovery and improved bladder bowel incontinence. At the time of discharge (10th postoperative day), patient regained power of 4/5 from 0/5 preoperatively, improved tone, sensory improvement by 80%, and regained bowel bladder control. At last follow-up of 2 years, patient recovered completely in terms of functional and neurological evaluation. Patient was walking normally with normal bowel bladder control at the last follow-up.

## Discussion

In 1869, first case of SSEH was reported by Jackson (reported by Fedor et al 3 ). Over 400 cases have been reported in the world literature with largest review of 107 patients of SSEH by Fedor et al. 3 This is more common in adult males with M:F ratio of 1.4:1 4 and commonly involves cervical–thoracic region.

Acute postpartum paraplegia commonly occurs due to predisposing factors, that is, trauma, coagulation abnormalities, vascular malformations, preeclampsia or iatrogenic, due to spinal needle for anesthesia during labor. When acute postpartum paraplegia occurs due to SSEH without any predisposing factors mentioned above, as seen in our case, is an extremely rare occurrence and not reported earlier in literature. SSEH in relation to pregnancy have been reported very few times in literature. 5 SSEH originates from venous blood or arterial blood is still a controversial issue with more inclination in literature toward acceptance of venous blood collection and resultant hematoma formation. 2 6 7 During pregnancy, hormonal changes may increase the fragility of vessel wall causing SSEH and may be the cause of acute postpartum paraplegia in our case. 8

In cases with acute postpartum paraplegia with bowel bladder involvement, SSHE should always be kept in mind as a cause of acute onset postpartum paraplegia. Other differentials to be included are epidural abscess, acute prolapsed intervertebral disc (PIVD), acute spinal ischemia, spondylitis, transverse myelitis, and dissecting aortic aneurysm. 9

MRI is the best modality to evaluate and diagnose SSEH. It helps in early diagnosis, accurate determination of haematoma size, level of involvement, adjacent spinal cord changes, and amount of cord compression so that patient can be managed in time and properly with surgical intervention if required 2 as soon as possible to improve the chances of neurological recovery. 10 But MRI characteristics which can predict the outcome of management don't exist in literature at present. 11

Commonly done procedure for SSEH includes complete laminectomy with hematoma decompression. We have done the same in our patient with excellent follow-up result. When SSEH occurs due to coagulation abnormalities or associated with mild neurological deficit or neurological deficit improving on its own, such cases can be managed conservatively with success. 12 In review of literature, we found that postoperative recovery after SSEH depends on preoperative neurological deficit and time between symptom onset and surgery. 13 14 15 Better prognosis found in cases associated with initial less severe neurological deficit and with shorter time to surgery from the onset of symptom. 13 14 15 16 We found excellent result in our patient as we have operated our patient within 48 hours from onset of symptoms.

## Conclusion

Acute onset postpartum paraplegia in a healthy female with no significant past history, predisposing factors or intrapartum factors may be caused by SSEH which is an extremely rare pathology during pregnancy/postpartum. Such cases must be managed on emergency basis as early and proper treatment has an excellent prognosis.
